# Supplementary material for: Empirical methods for controlling false positives and estimating confidence in ChIP-Seq peaks
Source: BMC Bioinformatics. 2008 Dec 5;9:523. doi: 10.1186/1471-2105-9-523 (PMC2628906; doi:10.1186/1471-2105-9-523)
Supplement: Additional File 1 — A variety of html documents from the USeq web site detailing the available applications, their best usage, output file type descriptions, command line menus, etc. [file 1471-2105-9-523-S1.zip › USeqUserGuides/JavaDoc.zip!/JavaDoc/bioroot/Search.html]

Search


|  |  |  |
| --- | --- | --- |
| **Overview** \| **Package** \| **Class** \| **Use** \| **Tree** \| **Deprecated** \| **Index** \| **Help** | |  |
| **PREV CLASS**   **NEXT CLASS** | **FRAMES**    **NO FRAMES**     **All Classes** |  |
| SUMMARY: NESTED \| FIELD \| CONSTR \| METHOD | DETAIL: FIELD \| CONSTR \| METHOD |  |


---


## bioroot Class Search

```
java.lang.Object
  javax.servlet.GenericServlet
      javax.servlet.http.HttpServlet
          bioroot.Search
```

**All Implemented Interfaces:**: java.io.Serializable, javax.servlet.Servlet, javax.servlet.ServletConfig

---

``` public class Search extends javax.servlet.http.HttpServlet ```

**Author:**
:   nix
    Search processing servlet

**See Also:**: Serialized Form

---

**Constructor Summary**

`Search()`


| **Method Summary** | |
| --- | --- |
| `static java.lang.String` | `blastResultToHTML(BlastResult res)` |
| `static java.lang.String` | `checkPlasmidDBFile(DBUtil bioRoot)`             Looks for an upto date blast database file. |
| `void` | `doGet(javax.servlet.http.HttpServletRequest request, javax.servlet.http.HttpServletResponse response)` |
| `void` | `doPost(javax.servlet.http.HttpServletRequest request, javax.servlet.http.HttpServletResponse response)` |
| `static java.lang.StringBuffer` | `makeBlastSummaryTable(BlastResult[] res)`             Returns a summary HTML table, hotlinked id, name, score, expect |
| `static java.lang.String` | `makeHashSelectStatement(java.lang.String table, java.util.HashMap rowScore, UserBean userBean)`             Makes an SQL SELECT statement using a HashMap. |
| `static java.lang.String` | `makeMultiFASTA(java.lang.String tableName, DBUtil bioRoot)` |

**Methods inherited from class javax.servlet.http.HttpServlet**

`service`

**Methods inherited from class javax.servlet.GenericServlet**

`destroy, getInitParameter, getInitParameterNames, getServletConfig, getServletContext, getServletInfo, getServletName, init, init, log, log`

**Methods inherited from class java.lang.Object**

`equals, getClass, hashCode, notify, notifyAll, toString, wait, wait, wait`

**Constructor Detail**

### Search

```
public Search()
```


**Method Detail**

### doGet

```
public void doGet(javax.servlet.http.HttpServletRequest request,
                  javax.servlet.http.HttpServletResponse response)
           throws javax.servlet.ServletException,
                  java.io.IOException
```

:   **Overrides:**: `doGet` in class `javax.servlet.http.HttpServlet`
:   **Throws:**: `javax.servlet.ServletException`: `java.io.IOException`

---


### doPost

```
public void doPost(javax.servlet.http.HttpServletRequest request,
                   javax.servlet.http.HttpServletResponse response)
            throws javax.servlet.ServletException,
                   java.io.IOException
```

:   **Overrides:**: `doPost` in class `javax.servlet.http.HttpServlet`
:   **Throws:**: `javax.servlet.ServletException`: `java.io.IOException`

---


### blastResultToHTML

```
public static java.lang.String blastResultToHTML(BlastResult res)
```

---


### makeBlastSummaryTable

```
public static java.lang.StringBuffer makeBlastSummaryTable(BlastResult[] res)
```

:   Returns a summary HTML table, hotlinked id, name, score, expect

---


### checkPlasmidDBFile

```
public static java.lang.String checkPlasmidDBFile(DBUtil bioRoot)
                                           throws java.lang.Exception
```

:   Looks for an upto date blast database file.

    :   **Throws:**: `java.lang.Exception`

---


### makeMultiFASTA

```
public static java.lang.String makeMultiFASTA(java.lang.String tableName,
                                              DBUtil bioRoot)
```

---


### makeHashSelectStatement

```
public static java.lang.String makeHashSelectStatement(java.lang.String table,
                                                       java.util.HashMap rowScore,
                                                       UserBean userBean)
```

:   Makes an SQL SELECT statement using a HashMap. Retrieves all ids in keys


---


|  |  |  |
| --- | --- | --- |
| **Overview** \| **Package** \| **Class** \| **Use** \| **Tree** \| **Deprecated** \| **Index** \| **Help** | |  |
| **PREV CLASS**   **NEXT CLASS** | **FRAMES**    **NO FRAMES**     **All Classes** |  |
| SUMMARY: NESTED \| FIELD \| CONSTR \| METHOD | DETAIL: FIELD \| CONSTR \| METHOD |  |


---
